# State-of-the-art MEMS and microsystem tools for brain research

**DOI:** 10.1038/micronano.2016.66

**Published:** 2017-01-02

**Authors:** John P. Seymour, Fan Wu, Kensall D. Wise, Euisik Yoon

**Affiliations:** 1 Department of Electrical Engineering and Computer Science, University of Michigan, Ann Arbor, MI 48105, USA; 2 Diagnostic Biochips, Inc., Glen Burnie, MD 21061, USA; 3 Department of Biomedical Engineering, University of Michigan, Ann Arbor, MI 48105, USA

**Keywords:** brain research, electrophysiology, MEMS, microelectrodes, neural engineering, neuroscience, optoelectrodes, optogenetics

## Abstract

Mapping brain activity has received growing worldwide interest because it is expected to improve disease treatment and allow for the development of important neuromorphic computational methods. MEMS and microsystems are expected to continue to offer new and exciting solutions to meet the need for high-density, high-fidelity neural interfaces. Herein, the state-of-the-art in recording and stimulation tools for brain research is reviewed, and some of the most significant technology trends shaping the field of neurotechnology are discussed.

## Introduction

‘Neuroscience today is like chemistry before the periodic table: People knew about elements and compounds but lacked a systematic theory to classify their knowledge.’ –Paul Allen and Francis Collins, Wall Street Journal, 2013 (Ref. 1).

The lack of a systematic theory of neural activity is complicated by the scale of the human brain, with an estimated 85 billion neurons, 100 trillion synapses, and 100 chemical neurotransmitters^2^. Understanding what makes any one neuron fire, or not fire, is a central question in neuroscience, and thus, the ideal sensing tool must span from the single neuron to its complex network of connections if we are to understand how a particular ‘cell type’ assimilates information^3,4^. In doing so, neuroscientists will identify new circuit ‘elements’ or neuronal cell types that may someday provide the world with a general theory of brain activity^5,6^, similar to how the periodic table arose from the study of repeating physical properties. Microelectromechanical systems (MEMS) and microsystems have enabled the study of neurons from the single unit to the scale of large populations, and all indications are that these technologies will continue to be an important tool-making platform for the neuroscience community.

Since the 1950s, recording capacity has been steadily increasing through the use of improved microelectrode technology, but this technology alone has not yielded the fundamental breakthroughs required to thoroughly understand the cellular components of a neuronal circuit. Beginning in 2005, seminal studies on optogenetics introduced methods for exciting and inhibiting neurons in ways specific to genetic and chemical markers of a particular cell type^7,8^. Optical control of genetically engineered ion channels is a powerful tool for parsing circuit elements and cell types in the dense heterogeneous populations surrounding a microelectrode recording site^4,9^. Biotechnologists are rapidly discovering new opsins (light-activated ion channels)^10–12^, and neurotechnologists are racing to scale light-delivery instruments, while simultaneously scaling electrical recording capabilities in the illuminated regions of tissue. Like optogenetics, other novel means of neuron control are beginning to be used, especially in the form of small molecules either ‘caged’ and released with local light stimulation^13^ or with receptor-specific ligands that can be controlled temporally and locally with other drugs (commonly called DREADDs for designer receptors exclusively activated by designer drugs)^14,15^. The contribution that microscale devices can make to these latest biology tools is still unproven but certainly intriguing given the many delivery, sensing, and actuation modalities that can be applied.

The potential to generate breakthroughs in mapping brain activity has prompted many governmental and non-governmental agencies to invest in new tools. The Human Connectome Project, an early mapping initiative, began in the United States in 2010 and emphasized macroscale anatomical connections. Europe’s Human Brain Project is a 10-year program that began in 2013 and increased the focus on supporting neurotechnology development for functional mapping in animals and humans. President Obama’s ‘brain research through Advancing Innovative Neurotechnologies’ (BRAIN) Initiative began funding research in 2014, with funding also tailored toward technology development for functional mapping and microscale neural circuit reconstruction. Japan announced their own program, Brain/MINDS, in October 2014, which is specializing in mapping activity in a marmoset animal model. An important aspect of brain mapping technology is that it should also be compatible with awake behavioral studies, which will require considerable advances in miniaturization and packaging. This surge in investment worldwide will have long-term benefits for all societies by improving the understanding of neurological diseases, which are the cause of 6.8 million deaths annually^16^. Beyond the obvious health benefits, insight into how animals and humans self-learn and perform pattern recognition will inform the burgeoning field of neuromorphic computing^17,18^. Neuromorphic computing is a biomimetic architectural approach that is replacing the von Neumann architecture with highly parallel, analog processing to achieve brain-like energy efficiency and adaptability. Systems neuroscience knowledge gleaned from neurotechnology will disrupt the microprocessor industry, while creating powerful new research tools and consumer products.

This paper will review recent developments in MEMS and microsystems for large-scale sensing and perturbation of brain activity with a focus on electrical recording and optical stimulation modalities. Electrical recording methods provide the greatest temporal resolution and frequency range and complement genetically targeted optical stimulation. New modalities also have the potential to contribute and perhaps displace electrical approaches in some applications. Two nascent implantable technologies include optical recording devices and optical stimulation, both used in conjunction with genetic modification to enable either an optical to ionic transduction or an ionic to optical transduction. Finally, despite modest improvements in microsystem density and integration in the last decade, we present encouraging trends in high-density circuit architecture and packaging.

## Recording brain activity

### Brief history

**Figure 1:**
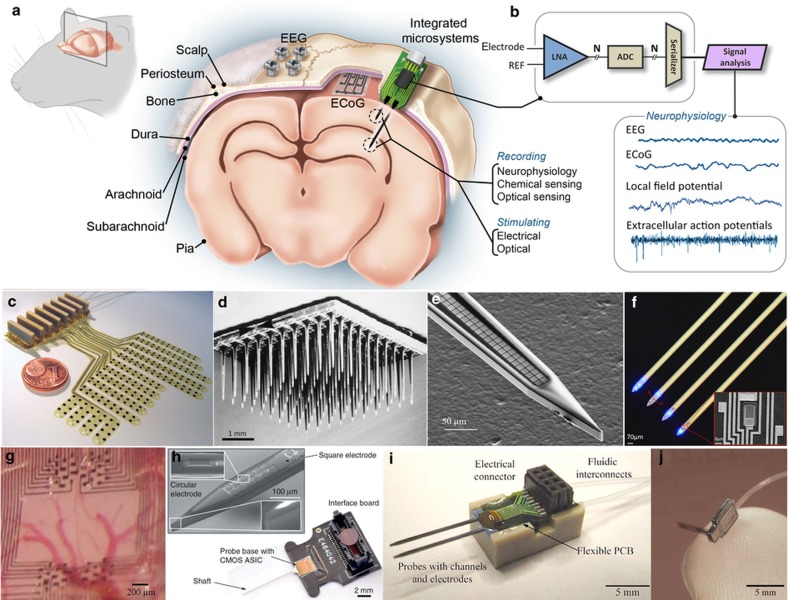
Recording and stimulating technologies vary across scale and degrees of invasiveness. (**a**) Illustration of the rodent brain and a variety of technologies from electroencephalogram (EEG) to intracortical microelectrodes. (**b**) High-density systems will increasingly require built-in active electronics to serialize large data streams and reduce the size of the connectors. Sample electrical signals show the amplitudes of various signal sources. The intracortical arrays are often microelectrodes but may also include chemical and optical sensors. (**c**) Polyimide electrocorticogram (ECoG) for large area mapping^67^. (**d**) A “Utah array” with 400 μm shank spacing and 100 channels has been used in human studies^50^. (**e**) Close-packed recording sites with 9×9 μm area and a pitch of 11 μm^178^. (**f**) MicroLED optoelectrode made from GaN on silicon^176^. (**g**) Parylene ECoG with greatly improved resolution over EEG and even single-cell capabilities^23^. (**h**) CMOS integration on probe shaft and backend^40^. (**i**) Fluidic probe for drug delivery^45^. (**j**) Active 3D silicon recording system with flexible parylene interconnect^182^.

The intracellular electrode was fundamental to understanding the action potential and remains the gold standard for understanding single-cell neurophysiology^19–21^, but it is difficult to scale and is damaging to the cell. The extracellular electrode, which can electrically record the signature of several neuronal action potentials, proved to be another major breakthrough because an array can sense a large number of single-neuron action potentials with limited disruption to the local circuit. The extracellular microelectrode (Figure 1) penetrates the brain and has a recording range of 65 (Ref. 22) to 150 μm (Ref. 23). Importantly, this electrode can capture ‘single-unit activity’ or ‘spikes’ in the context of population dynamics and thus was the first technology capable of circuit mapping^24^. Microwires were the first such devices and have been an effective research tool for studying the brain for nearly 60 years (Refs. 25,26). Microwires evolved into stereotrodes and tetrodes^22,27^ with a variety of insulating and conductor materials depending on the recording or stimulation requirements. Electrode measurements approximate the superposition of voltages from a series of monopolar current sources in the local tissue, derived from Ohm’s law,

(1)V=∑i=1nIi4πσri

where *I*_*i*_ is the current of one point source, *σ* is the conductivity of the extracellular space, and *r*_*i*_ is the distance from the source to the electrode (a dipole assumption results in a more complex isopotential and an amplitude proportional to 1/*r*^2^, but arguably the ratio is neither a monopole or dipole^28–31^). Given the close spacing of tetrodes, multiple spike signals can be used to triangulate and localize a specific cell in space^32^.

The pioneering work of Wise at Stanford University^33,34^ and at the University of Michigan^35,36^ in developing microfabricated silicon electrode arrays was a major advancement. The geometrical precision of lithographic techniques has allowed neuroscientists to imagine unique electrode designs having unprecedented site density. In 1982, engineers first used selective boron doping of microelectrode silicon probes to create wet etch-stops in a technique that produced smooth needle-like structures ideal for minimizing tissue damage^37^. This technique spread throughout the MEMS community and formed the basis for many other novel electromechanical structures. The planar lithography approach has evolved over the years to include integrated interconnects^38^, active electronics^35,39,40^, cochlear implants^41,42^, polytrodes^31^, and three-dimensional arrays^29,43–45^. An important simplification for defining and releasing fine neural probe structures has been the use of silicon-on-insulator (SOI) wafers and deep reactive ion etching (DRIE)^46,47^ that many groups have adopted.

Another MEMS technology that has redefined the microelectrode is the ‘Utah’ array, originally developed by the Normann group at the University of Utah^48–50^. The ‘Utah’ array is generally fabricated as a 10-by-10 array of tines machined from 3-mm-thick silicon wafers. These tines are anisotropically etched and doped to form a monolithic array of conductive needles. Lithography is used on the backend to define bonding pads with one channel per tine. This approach provides robust mechanical properties that have made it very popular in primate research^51–54^. This silicon electrode platform has been the basis for successful human trials using a brain machine interface^55–57^ and has been adapted by several groups for wireless integration^58–60^.

Another form of high-density recording arrays is surface arrays, either for *in vitro* or *in vivo* experiments. Surface arrays for tissue slices and retinal recording are known as multi-electrode arrays (MEAs). Electrical recording with MEAs from the retina or hippocampal slices has provided the highest density of information available, in part because there is no requirement for miniaturization on the backend. Despite the lack of physical constraints, certain MEA architectures have achieved unprecedented miniaturization, as discussed in Subsection ‘High-density active recording’ below. Commercial MEAs containing tens of thousands of electrode channels provide remarkable resolution of neural activity spreading over time, often referred to as electrical imaging, and have been designed for tissue culture and slices. MEAs are usually CMOS devices with relatively simple post-CMOS metallization. Several reviews on *in vitro* or MEA approaches are available^61–63^. However, nanoscale MEMS-based processing is increasingly being developed to achieve intracellular recordings in particular^64–66^. We will return to the topic of MEAs because advances in wafer thinning, chip integration, and flexible electronics are blurring the line between *in vitro* and *in vivo* devices.

Surface recordings on the brain record an electrocorticoencephalogram (ECoG) and are often referred to as ECoG arrays or microgrids. These arrays are effectively flexible versions of MEAs. ECoG arrays are less invasive than microelectrodes and have higher spatial resolution^67^ than electroencephalogram (EEG) arrays, which is limited to signals spatially filtered by the dura and skull (Figure 1). Macroscale versions of an ECoG array are generally platinum discs soldered to metal wires and molded in medical grade silicone or polyurethane. These devices exist commercially, but a significant push toward microscale structures has revealed important physiological data that neuroscientists have embraced^23,68^. Advancing ECoG and EEG electrodes and their microsystems will be particularly useful for conducting neuroscience and neurology studies in human patients.

ECoG and microECoG are the best surface array methods for source localization, but recent advances in EEG source localization when accounting for patient-specific anatomy and conductivities now claim sub-cm^2^ resolution^69^. Technology improvements for EEG systems have also witnessed an increase in funding, including those made at the Army Research Laboratory in the United States, with the goal of making EEG a practical tool for widespread human-based neuroscience in real-world applications^70^. EEG systems will benefit from microscale electrode features such as microneedle electrode designs that reduce the variability in skin contact^71^ and mixed-signal front-ends with reduced size and power to eliminate long analog wires.

### Advantages and challenges of high-density recording arrays

Systems neuroscience is seeking to monitor single-neuron activity in the context of very large populations to identify how the constituent parts lead to the emergent properties of the whole. The number of simultaneously recorded neurons has been doubling approximately every 7 years (Refs. 72,73). Additional investment in brain mapping technology is ongoing and justified because even at this rate of doubling we are still far from achieving recording densities capable of whole-brain mapping^6,74^. Although new optical imaging and recording modalities will certainly accelerate this rate of discovery, electrical recording methods still provide the greatest temporal resolution and frequency range. Many neuroscientists continue to rely on the simultaneous measurement of single-cell spiking and local field potentials (which includes delta, theta, alpha, beta, spindle bursts, and gamma oscillations) to derive complex network effects^75^. Tetrodes (four wires closely spaced together) continue to be the workhorse of electrophysiology because they are often fabricated in research labs at low cost. However, neuroscientists have increasingly found it efficient to use high-density microfabricated electrodes. Microfabricated arrays offer a large design space and geometric precision and can at least match the two-dimensional cellular density of the brain over a greater span than that observed for tetrodes. Three other compelling advantages of microfabricated recording arrays are also worth noting. First, overlapping recording regions can form multiple tetrodes or polytrodes and have proven the best means for maximizing single-cell identification on a per-channel basis. Microstructures of various materials and geometries also offer ways to minimize adverse tissue reactions. Finally, the integration of MEMS-based probes with actuators and amplifier microsystems will provide more effective tools for brain mapping (discussed in Sections ‘STIMULATING BRAIN ACTIVITY’ and ‘SCALABLE IMPLANTABLE MICROSYSTEMS’, respectively). Despite all of these advantages, several key remaining challenges should be addressed.

The recording of spikes in a mammalian brain is as much a software challenge as it is a hardware one. Thermal, electrical, and biological noise sources can combine to severely limit the signal-to-noise ratio^76,77^ and, when combined with spiking variability, can result in false spike detection, missed detection, and erroneous classifications^78,79^. Despite >50 years of advances and a plethora of algorithms for addressing issues of accuracy and speed, neuroscientists, mathematicians, and technologists are still far from reaching the goal of real-time automated spike sorting^30,80^. The stereotrode^27^ and tetrode^22^ were hardware solutions designed to help address the problem of spike sorting accuracy. Overlapping recording regions to maximize confidence in a putative action potential was first recognized using two twisted microwires measuring 20 μm in diameter and spaced only a few microns apart^27^. In one study, a MEMS-based array with a linear tetrode was validated using intracellular recordings as ‘ground truth’ for spike sorting. Tetrode versus single-site recordings yielded both greater accuracy and greater numbers of units per channel^78^. Another study extended this logic and used a dense 2D array of 54 channels on 1 shank to compare the performance of virtual tetrodes (grouping four adjacent electrodes) and polytrodes (more than four electrodes). The authors’ data were limited to relatively large site pitches (43–65 μm) but showed that polytrodes outperformed even tetrodes in isolating multi-unit clusters into individual single units^31^. This same study also found as many as 24 single units in a virtual tetrode in the relatively sparse visual cortex. More recently, software algorithms that utilize precise spatial information as part of the sorting logic show great promise for improving both speed and accuracy^30,80–82^. Investigating the optimal microelectrode size and pitch for speed and accuracy in spike sorting is an important research area.

Accurate real-time automated spike sorting is still unproven but will be a heralded breakthrough in systems neuroscience and in clinical applications such as neural prostheses requiring a brain-machine interface. Using MEMS-based arrays to improve software tools for neuroscientists also involves neuronal location information^9,24,29,31^ and cell classification of neurons^4,83^, which are critical in brain mapping. The need for technologies to enable cell typing in brain research was highlighted as the number one priority by the U.S. BRAIN Initiative^84^, and combining recording techniques with genetically targeted methods such as optogenetics and pharmacogenetics^85^ is expected to make cell typing more reliable^6^. MEMS-based devices can certainly provide the resolution if this expectation proves to be the enabling requirement for new software tools, but device reliability and micro-sized packaging solutions are significant constraints that must be part of the solution.

### Tissue response and structure size

The adverse tissue response to implantable neurotechnology has been an ongoing area of active research and is reviewed elsewhere (Jorfi, Capadonia 2015)^86^. Important components of the adverse response have been studied, including insertion trauma^87–89^, the intrinsic foreign body response^90,91^, and strain-induced damage from mechanical mismatch^92–94^. Long-term reliability is clearly more of a challenge in clinical applications than for neuroscience; nonetheless, tools for brain mapping should be designed to minimize disruption to the circuits being investigated. The size of the device placed into spinal cord or brain tissue undoubtedly affects the degree of initial damage. The mean distance from the center of a neuron (somatic center) to the nearest microvasculature is only 15 μm (Ref. 95); thus, regional damage to the blood–brain barrier is unavoidably a function of size^88^. The intrinsic foreign body response is complex and intertwined with the issue of micromotion, which is related to mechanical factors such as the probe cross-sectional area, lattice or porous architecture, total surface area, and stiffness. Biochemical factors may include material stability, chemical properties, and protein adhesion. Work on these mechanical factors will be briefly reviewed here because MEMS allow for the selection of a large range of materials and geometries.

**Figure 2:**
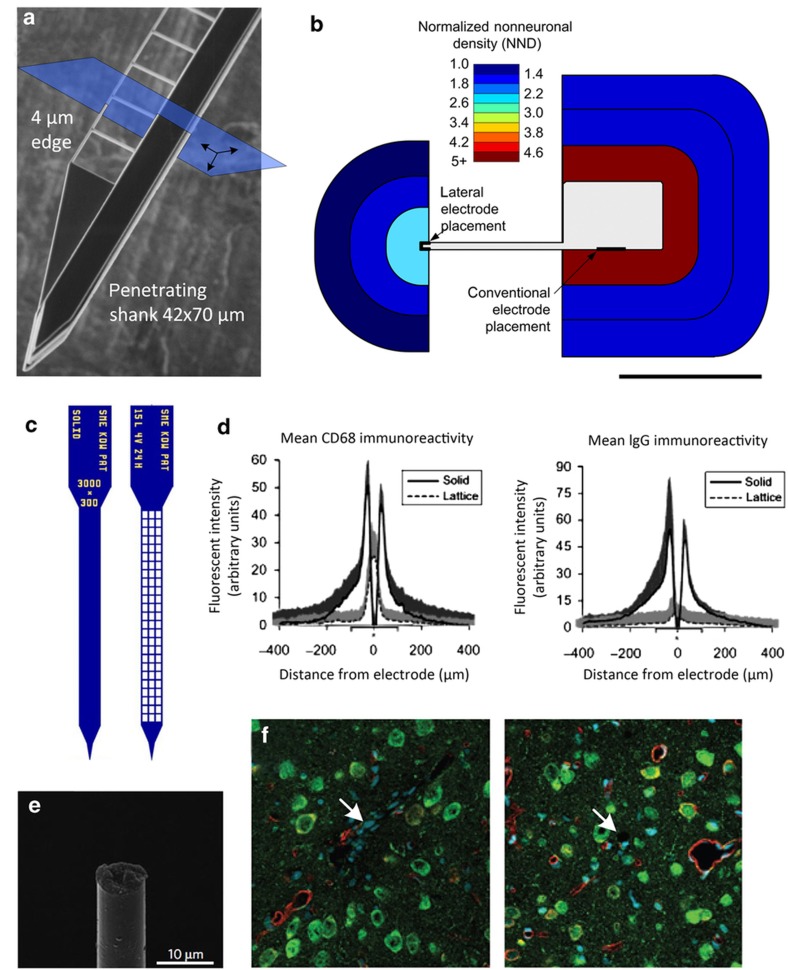
Seminal work supporting the hypothesis that the tissue response is a function of local device structure. (**a** and **b**) Tissue around the end of a thin polymer structure showed significant reduction in encapsulating cells (modified from Ref. 98). (**c** and **d**) Tissue response around solid and fine lattice structures showed significant reduction in reactive markers such as CD68 and IgG^99,104^. (**e** and **f**) Carbon fiber microthreads with an 8-μm diameter reduced tissue reactivity and improved neuron density of microthread^101^. IgG, immunoglobulin G.

There is mixed evidence indicating that geometric size is an important design criteria in achieving reliable high-density recordings (Figure 2). Evidence from several groups indicates that when small features are used there is a significant improvement in many histological markers, but whether those are the best markers to predict performance is unclear. In early studies on geometry, differences in relatively large structures resulted in similar long-term outcomes^96,97^. When cellular-scale probe thickness dimensions (5 μm) were compared against the dimensions of a polymer shank (~50 μm), however, there was a significant difference in non-neuronal density (300% higher at the shank) and neuronal density (40% lower at the shank) in favor of smaller structures^98^ (Figure 2a). Qualitatively, the interface around the 5 μm edge also showed improvement in microglia and astrocyte reactivity. Two other studies independently supported these results using different materials and elastic moduli, with similarly small feature sizes^99–101^. Silicon was used to make a lattice probe with exceptionally fine features^102,103^ measuring 5 μm that was implanted and reduced a variety of glial reactive markers^99,104^. The most creative application of the lattice structure was recently demonstrated by creating an injectable SU-8-based lattice inserted through a 22-gauge needle and electrically connected to a printed circuit board (PCB) using anisotropic conductive film during surgery^105^. Although histological evidence supports the use of smaller, lattice-like structures, no group has yet provided strong evidence that reliable single-cell resolution can be achieved across many channels for more than even one year. Currently, the gold standard among neuroscientists seeking long-term single-cell recording is the use of a microdrive to periodically ‘tune’ the position of silicon neural probes (particularly those with thicknesses ranging from 12 to 15 μm and shanks measuring approximately 60 μm wide for rodent studies)^106^. Therefore, interest in and funding for advanced microelectrodes have been increasing given both the current success and the potential for further improvements of large-scale long-term electrophysiology.

### Substrate materials and microfabrication

**Figure 3:**
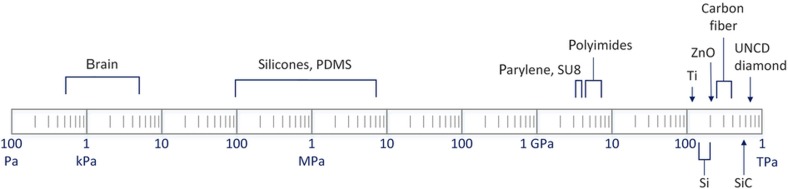
Log scale of elastic modulus for many substrates used in implantable arrays. Seminal research has covered inorganic substrates such as silicon^33^, titanium^107^, diamond^108^, zinc oxide^109^, and silicon carbide^110^. Studies on organic substrates have covered carbon fiber^101^, parylene^111^, SU-8^105,113^, polyimide^114,115^, and silicone^116^.

A variety of materials and methods have been explored for use in neural probes. Figure 3 highlights seminal studies on novel substrates and compares the substrates’ intrinsic stiffness. Each of these substrate materials has shown good biostability, albeit with varying degrees of evidence, but many other properties must also be considered in the context of the application at hand, including the practical processing questions regarding deposition, etching, adhesion, and general process compatibility. Many substrate materials have been explored to date, providing researchers an excellent toolbox.

Substrates can be classified as inorganic (for example, silicon^33^, titanium^107^, diamond^108^, zinc oxide^109^, and silicon carbide^110^) or organic (for example, carbon fiber^101^, parylene^111,112^, SU-8^105,113^, polyimide^114,115^, and silicone^116^). Silicone, which includes polydimethylsiloxane (PDMS), is an important class of silicon-based organics that provides a useful range of properties, particularly elasticity, not otherwise available. Brain tissue elasticity is still two orders of magnitude lower than the softest PDMS currently being used (not including Ecoflex® or gels, Macungie, PA, USA). Nonetheless, useful devices have been demonstrated on either end of the modulus spectrum, with stiffer materials enabling robust micron-sized structures and elastic materials enabling stretchable substrates at the expense of thickness or requiring the use of insertion aids. PDMS, the best known of the silicones, has proven amazingly versatile in the microfluidics and medical device communities but has found limited use in thin-film devices. The primary challenges have been metal adhesion and creating thin substrates, although improvements are forthcoming^117,118^. Important work reported by Minev *et al.* (Ref. 116) recently demonstrated that PDMS is an effective substrate for stimulation and drug delivery. As the feature size improves, the material may become equally useful for high-density recording. Even more under-utilized than PDMS are polyurethanes, polymers with urethane links (NH–(C=O)–O–), which may also prove important because their mechanical properties are highly tunable and their surfaces are readily functionalized^119,120^.

Reducing the mechanical mismatch between a MEMS device and the extremely soft brain^121,122^ (Figure 3) has been a focus of some research. The challenge is to insert a highly flexible substrate along a straight trajectory to a deep brain region. In addition, an insertion aid that has a sharp, rigid tip is necessary to pierce the dura or pia matter of the brain (Figure 1). One approach involves the creation of a biodegradable stiffener using silk over a thin parylene-C structure and shaping the silk with a microfabricated mold^123^. As indicated by Equation (2) (rectangular cantilever stiffness),

(2)k=Ewt34ℓ

lowering the Young’s modulus *E*, the width *w*, and particularly the thickness *t* should greatly reduce tethering forces. Several groups have tried to quantify the local strain effects using finite element models^92,124^. Nonetheless, no research has clearly identified a threshold of relative stiffness that will mitigate the tissue response or, more importantly, enhance the recording quality. Silicones not only reduce the mechanical mismatch but achieve the greatest yield strain of all substrates used to date. Creating metal conductors that are resilient inside a stretchable substrate utilize wavy metal conductors^125,126^, thin metals forming percolation networks^116,127^, organic conductors^128,129^, and stitched gold wires^130^. Further development of silicone or polyurethane substrates requires improving the feature resolution of the conductors and structures, validating the long-term adhesion and insulation of the conductors to the substrate, and engineering higher density packaging options.

An application in which flexibility and fracture toughness are absolute necessities is the electroencephalogram (ECoG). In this case, a microgrid is placed over the curved surface of the brain that requires close contact with the surface to maximize the recorded signal amplitudes. An early design used a polyimide substrate measuring 20 μm thick and had an electrode spacing of 1 mm (Ref. 68). Others have further improved the flexibility by developing a polyimide array measuring only 2.5-μm thick supported by biodegradable silk to aid in placement^131^. Most recently, the Buzsáki group developed the ‘NeuroGrid,’ a 4-μm-thick parylene-C ECoG array featuring low-impedance electrodes and a pitch of 30 μm to enable tetrode sorting techniques, resulting in the ability to record single-cell activity in both rats and humans^23^ (Figure 1g). This latest development is particularly significant because it overturned the long-standing assumption that single-unit recordings were only possible with intracortical microelectrodes.

By far, the most successful material for intracortical extracellular neuroscience research has been silicon, which has led to at least four neural probe companies at the time of this writing. Some of most advanced silicon designs include double-sided, high-density arrays^29,102^, three-dimensional arrangements^43^, and integrated electronics^39,40,132^. Although the inherent brittleness of silicon is a concern for some clinical applications, its electrical, mechanical, and thermal properties offer the greatest design options and are supported by a wide range of commercial microfabrication tools. The large mechanical stiffness of silicon has been cited as a possible hindrance to long-term recording quality, but achieving thinner and finer structures would resolve this concern in many situations. Silicon devices can penetrate many brain types and depths. Unlike polymer arrays, silicon and other stiff materials have the ability to be moved on a microdrive during long-term recording and thereby maximizing the number of cells recorded in a session^106^.

Other stiff materials include ultrananocrystalline diamond^108^, SiC^110,133^, and particularly carbon fiber materials^101,134^. These materials are all relatively new to field but have two significant advantages over silicon—a higher fracture toughness and a higher elastic modulus. These substrates may someday prove to be more reliable and achieve smaller dimensions in the hands of neuroscientists, but first manufacturing and handling limitations must be addressed.

### Advanced electrodes

The electrode recording site is ultimately the interface where cellular activity is accessed and where some improvements must continue to be made. Sites are usually at least 10 μm in diameter, but the real tissue interface occurs at the scale of the electrical double-layer (Helmholtz layer), which makes all electrode technology from the EEG scale to the single-cell scale fundamentally a nanotechnology challenge. Several extensive reviews provide a historical perspective of this research space^135,136^. Electrical stimulating and recording electrode requirements share some similarities, but the electrochemical stability and current injection requirements of stimulation demand greater rigor in material selection and validation. Attention to charge-carrying capacity, charge balancing, voltage limits due to water hydrolysis, and long-term testing is required for function electrical stimulation, which continues to make important contributions to neuroscience^137^.

Electrode requirements for recording have evolved as scientists have debated the optimum site impedance and size. Lempka *et al.* modeled the effect of electrode size on signal amplitude and showed that site diameters ranging from 7.5 to 20 μm produced nearly identical amplitudes; however, large pyramidal cells were assumed as current dipoles^138^. The results may be very different when recording near apical dendrites or smaller cell types. When site sizes >20 μm can be used, thin-film materials such as Au, Pt, and Ir often show good performance. For tetrode sorting techniques discussed above, a pitch (diameter plus gap) as small as 20 μm is desirable. In these applications, thin-film Au, Pt, and Ir would all produce significant noise without additional steps to increase surface roughness. Research has demonstrated that electroplated Au^29^, reactive sputtering of TiN^139^, sputtered iridium^140^, and activated iridium^139^ all lower the electrode impedance and decrease the electrical noise. Modification of the electrode material or the deposition method is a well-proven means to lowering the impedance and therefore the thermal noise. This modification is also likely to lower susceptibility to electromagnetic interference^76,141^.

New electrode alternatives continue to be developed and tested because past methods either do not provide sufficiently low noise or are unstable over time *in vivo* (or may provide insufficient charge storage capacity for electrical stimulation). Delamination and stability of the coating is a serious challenge. Causes include mechanical and chemical instabilities that are a function of the deposition method or due to non-reversible redox reactions. Furthermore, after implantation, the electrode will immediately biofoul and undergo electrochemical interactions. Over the last decade, conductive polymers, particularly poly(3,4-ethylenedioxythiophene) (PEDOT), have received much attention, outperforming thin-film metals in the first several weeks of use before biofouling and the foreign body response presumably reduce performance^142,143^. PEDOT has excellent charge injection capacity^144^ and one of the lowest site impedances per unit area of any material. The two most common dopant molecules for PEDOT are polystyrene sulfonate (PSS) and, more recently, carbon nanotubes (CNT)^143^. Nevertheless, no electrode material has proven to monitor single neurons for years or even months without significant degradation in the signal-to-noise ratio; thus, more research on the electrode-tissue interface is certainly needed.

Regardless of which advanced material may solve this problem, the manufacturing method chosen is an important part of development. Some common electrode materials for brain research, for example, electroactive iridium, electroplated gold, and electrodeposited PEDOT, are deposited after the electrode array is in the final assembled state. This approach creates efficiency and uniformity challenges that could be better addressed at the wafer level, for example, batch electrodeposition. A cost-effective solution recently demonstrated is the use of a dry etchant to roughen thin-film gold at the wafer level^145^, resulting in impedance values similar to those of PEDOT. Methods such as this may prove to be effective to neuroscientists and efficient to neurotechnologists.

## Stimulating brain activity

### Introduction to optogenetics

Recording passively from local brain circuits is informative, but neuroscience can be much more effective at mapping a circuit by using controlled stimulation while monitoring cellular responses and their correlation to animal behavior. Over the past few decades, the electrical stimulation of the brain has provided tremendous insight into its functionality^146,147^. However, advanced neuroscience capable of studying cellular interactions in complex networks can be accelerated with selective activation or silencing of neurons of specific types. This feat cannot be achieved easily by electrical stimulation due to its lack of spatial resolution, non-specific stimulation, and the inability to silence neurons^148^.

Optogenetics has begun to improve neuronal circuit analysis by introducing photosensitive proteins (opsins) into specific cell types such that these cells can respond to an optical stimulus with defined action potential patterns^8,149^. Using an appropriate wavelength to target a particular opsin(s), cell-type specificity can be achieved with well-controlled spatial and temporal resolution (on the order of milliseconds)^7^. For example, channelrhodopsin-2 (ChR2) and halorhodopsin can be co-expressed in the same cell types for the depolarization and hyperpolarization of this specific target using blue light (~473 nm) or yellow light (~590 nm), respectively^7,150–154^. This specific targeting allows for more sophisticated manipulations of neural activity and the testing of spike timing during specific neural computations and behaviors, but the sophistication is also a function of the light delivery tool itself. A major trend in optogenetic stimulation is the improvement of spatial selectivity because illuminating large volumes of tissue introduces a number of potential confounds to the experiment. There is the possibility of altering the threshold of excitation or creating action potentials because of light absorption and heat^155^ and the superposition of multiple spike waveforms on recording channels^156^. Furthermore, stimulating many neurons in synchrony is not a natural way to generate synthetic input^157^; therefore, we discuss several technological approaches that can address these limitations. Two-photon stimulation techniques offer the greatest spatial resolution^158^ and a large field of stimulation, but because it is not capable of accessing deeper tissue structures and requires head-fixed experiments, there continues to be great interest in improving MEMS-based optical stimulation devices.

### Early development of optogenetic tools

Despite recent rapid advances in optogenetics, supporting technologies for reliably delivering light to and record electrical signals from deep brain structures are not readily available. Early work involving *in vivo* optogenetics relied on the manual assembly of commercially available components such as microwires and optical fibers, which are not only bulky but can also experience large misalignments due to human error^9,159^. Since then, engineering efforts have gradually evolved towards MEMS technologies for miniaturization, high-density integration, and precise definition of the probe dimensions with lithographic resolution. For example, MEMS dielectric waveguides fabricated on silicon substrates for stimulating the brain at multiple locations with blue and red light have been reported^160^. However, no recording electrodes were integrated on these devices; therefore, they could not support both optical stimulation and electrophysiology. Stark *et al.*^9^ reported a hybrid approach for manually assembling optical fibers onto MEMS recording probes. Coupled to laser diodes of various emission wavelengths, this device could excite and silence neural populations monitored by a high-density electrode array^161^. Nevertheless, the manual attachment (gluing) of fibers to each probe shank is very labor-intensive, resulting in potential misalignments and contamination of the recording sites by misplaced glue.

### MEMS optical waveguide integrated probes

Advanced MEMS technologies can enable micron to sub-micron-scale features to be accurately defined using lithography. Planar architectures such as the ‘Michigan style’ probe shown in Figures 1e and f are particularly attractive for the integration of optics because of the versatility in depositing and patterning additional layers to form high-density optical and optoelectronic components.

**Figure 4:**
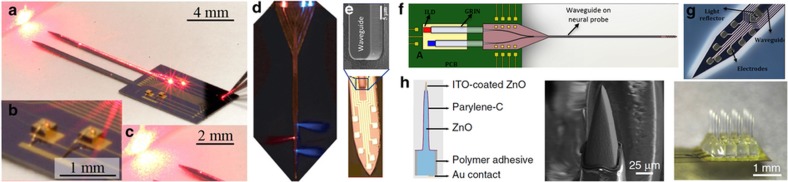
Example optoelectrodes with integrated waveguides: (**a**–**c**) Laser diode coupled waveguide probe demonstrating diode directly mounted on neural probe^165^; (**d**) a digital micromirror directing multi-color light into waveguides terminated with metal-coated corner mirrors^171^; (**e**) single waveguide on a silicon recording array^162^; (**f** and **g**) schematic of multi-color laser diodes coupled from a PCB using graded-index lenses and mixed on the neural probe and micrograph of an actual device^166^; and (**h**) a 4x4 ZnO array demonstrating a very similar form factor as the Utah array with the added capability of optical stimulation through the ZnO tine and ITO-coated tip^109^.

Some of the first neural probes monolithically integrating both optical and electrical components are illustrated in Figures 4a–g. In these devices, an optical waveguide is integrated onto the probe shank to deliver light from an externally coupled optical source to a stimulation site at the center of an integrated electrode array^162^. The waveguide can be made out of polymers such as SU-8 (Refs. 163,164) or dielectrics^162^ and has a small rectangular light emission area on the order of 100 μm^2^. The lithographically defined waveguide has a precisely positioned stimulation site relative to the recording sites to provide the spatial resolution necessary for circuit mapping (Figure 4e). The waveguide can also be freely configured to guide light through different paths^164^ or to have multiple stimulation sites^160,163^ for specific application needs (Figure 4d). The probe shank dimensions are defined with micron resolution using a double-sided DRIE process on an SOI wafer. Even a simple design having a single waveguide and eight electrode sites can demonstrate the utility of optogenetics from a high-density population such as the CA1 pyramidal layer in a rat by optically inducing single-unit activity from two cell types, which can be distinguished by the relative timing between the induced spikes and the light stimuli^162^.

An important modification to the waveguide approach is eliminating the need for a tethered optical fiber because it can severely limit animal movement, especially when multiple fibers are used. A recent report demonstrated the feasibility of coupling a bare laser diode chip to an integrated waveguide^165^. The semiconductor light-emitting device requires only thin flexible cables for power, which is attractive for freely behaving animal experiments. Using unpackaged bare laser chips has the advantage of efficient coupling to waveguides with a similar numeric aperture (Figures 4a–c) and reaching optical intensities of up to 29.7 mW mm^−2^ using a red (650 nm) laser diode. Given the high cost of laser diodes and alignment and packaging, it may be useful to package the source at the PCB and either butt couple or focus the source into the probe. A recent example of this approach used commercially available gradient refractive index lenses to couple end-fire lasers^166,167^ (Figures 4f and g). Another solution was demonstrated with a novel substrate material, zinc oxide, to form both the recording channel and waveguide in a form factor and fabrication method similar to the Utah array^109^ (Figure 4h). Both the on-chip and on-PCB approaches will undoubtedly be useful, but as one scales the number of independent light sources or stimulating at higher duty cycles, the on-PCB approach may be the better choice, as evidenced by thermal modeling. In contrast, coupling an light-emitting diode (LED) to a waveguide is a fundamentally inefficient task due to the Lambertian emission profile of LEDs and the small area of minimally invasive waveguides.

The equations governing the basic design constraints of coupling a light source to a waveguide and then coupling the light into tissue are highly dependent on material and geometry. To briefly summarize, thin-film approaches allow engineers to maximize irradiance in the tissue using the equation

(3)I=Ps⋅ηcoupling⋅ηscatter⋅Φgeometry

where *P*_*s*_ is the optical power of the source, *η* is the coupling efficiency or scatter efficiency, and *Φ* is the function accounting for geometric spread as a function of depth, waveguide radius, and numerical aperture^168^. By careful selection of materials and the waveguide geometry, the optoelectrode can be chosen to match the refractive index at each optical interface to maximize coupling, but it is also desirable to maximize the numerical aperture of the waveguide to accept a wider angle of incoming light and to emit a wider angle at the output using Equation (4),

(4)NA=n0sinθa=(ncore2−nclad2)1/2

where *n*_0_ is the surrounding refractive index and *θ*_*a*_ is acceptance angle. These factors are also related to the index of the core and cladding. Furthermore, the maximum efficiency in source-coupled waveguide is given by Equation (5),

(5)Ec=AWGNA2/(Asourcen02)

where *A*_WG_ is the cross-sectional area of the waveguide and *A*_source_ is the area producing light at the emitter. Even a small LED (100×100 μm) will have a source area, *A*_source_, ~10 000 times larger than that of a typical laser diode. On the far side of the waveguide, light will enter the tissue, and the effects of geometric spreading and light scattering inside the tissue create some constraints on the volume of tissue that will be irradiated. The first is the spreading angle. Equation (4) can also be used to calculate the divergence angle of light in the tissue, but in that case *n*_0_*=n*_tissue_. Unlike geometric spreading, scattering is a function of the wavelength of light^168^. Calculating the volume of excitable tissue also requires that some assumptions be made. First, one must estimate the irradiance threshold at which a neuron can be stimulated or silenced, which is a function of the opsin type, the consistency of expression in a cell, and local neuron orientation^9,169^. A second assumption must be made about the maximum irradiance allowed in tissue before heating^170^ and cellular^155^ changes occur. A Matlab tool for predicting the tissue irradiance and the heat generated using different spatial and temporal light input was provided by Stujenske *et al.*^170^. Despite these limitations, there is plenty of room for customizable illumination patterns given our control of numerical aperture, waveguide dimensions, and the addition of micromirrors^160,171^ and diffusion elements. Photonic waveguides are commonly fabricated with micron dimensions, and thus, the number of independent light ports will increase rapidly, especially as the light sources are more efficiently packaged and coupled at the device backend.

### MEMS LED integrated probes

An alternative to using a waveguide to transmit light from an external source to the probe tip is to integrate the light sources onto the probe tip directly. InGaN LEDs are attractive for optogenetic applications because the emission wavelength can be tailored for the activation of common opsins^172^.

GaN-based LEDs are most commonly grown on sapphire or SiC substrates for minimal lattice mismatch at the GaN-substrate interface^173^ because this structure enables efficient electron-to-photon conversion. Recently, LED arrays fabricated from a sapphire substrate have been demonstrated for optogenetics^174^. Thermal and optical characterization shows that blue LEDs can deliver enough optical power to activate ChR2 without overheating the tissue. However, no recording electrodes were integrated with these optical sources, and the probe shanks were not released from the sapphire wafer. The report demonstrates that the probe shanks can be patterned by laser dicing and that the substrate can be thinned mechanically; however, this approach may not achieve a needle-like probe body sufficient for fine features with minimal tissue damage.

**Figure 5:**
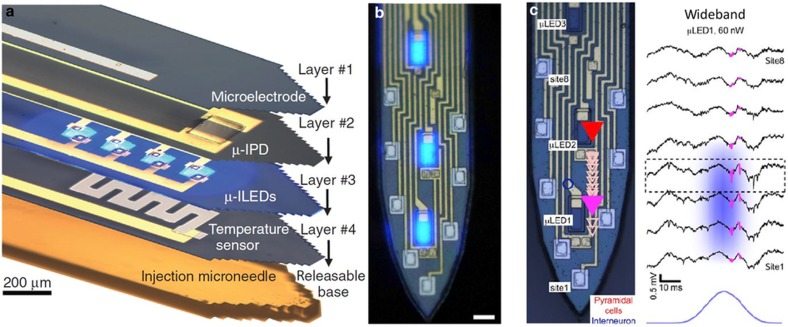
Fiberless optical stimulation using μLEDs. (**a**) GaN μLEDs grown on sapphire wafers and transferred onto a polymer substrate by laser-liftoff achieved 50×50 μm^2^ μLEDs^175^. (**b**) First demonstration of monolithic integration of multiple GaN μLEDs on silicon neural probes and capable of a 50 μm pitch. Scale*=*15 μm. (**c**) *In vivo* demonstration of same optoelectrode controlling pyramidal cells (PYR) in distinct parts of the CA1 pyramidal cell layer^176^.

To circumvent the difficulty in patterning conventional substrates such as sapphire, LEDs originally grown and patterned on sapphire can be transferred to another substrate by a laser lift-off technique. Figure 5a shows the assembly of several microfabricated LEDs at the tip of a flexible polymer platform for light generation precisely at the stimulation sites^175^. This approach is highly versatile, allowing for the integration of not only LEDs of different colors but also photodiodes and temperature sensors in a multi-layer structure to monitor the performance of the LEDs. Although the individual LEDs could be made relatively small (50×50 μm), the hybrid assembly resulted in a probe shank width of over 400 μm. Once implanted, the LEDs can be wirelessly controlled.

Our group’s ongoing work on the monolithic integration of μLEDs and electrodes onto silicon probe shanks is highlighted in Figures 5b and c. This highly scalable approach allowed for 12 μLEDs and 32 electrodes to be integrated onto a four-shank probe, where each shank was only 70 μm wide^176^. The μLEDs and electrodes have been defined to have cellular dimensions, which can provide high spatial resolution for single-unit stimulation and recording in a highly populated brain region. The biggest challenge to any μLED approach is reducing μLED crosstalk, often called stimulation artifact. Addressing this challenge will require improved EMI immunity and better system integration at the backend.

We reviewed two broad approaches to fiberless optical stimulation: the use of an optical waveguide to transmit light from an external source and LEDs integrated next to the recording channels. The waveguide approach affords the ability to choose from commercial light sources, with many available wavelengths and output power levels available. Coupling into the waveguide is greatly improved with the use of advanced packaging tools such as a die bonder. Furthermore, the electromagnetic interference and heat generated by the light source are more easily managed given the greater distance from the susceptible recording channels. In contrast, the direct integration of light sources on the probe shank allows for highly efficient coupling into tissue and creates a stimulation zone well matched to the recording zone. The LED efficiency is closely associated with the semiconductor-to-substrate interface defect density. Thus, the light-emitting materials and substrate are constrained, limiting the available wavelengths and process compatibility. Obtaining a reasonable power efficiency is much more critical for this approach than for the waveguide approach because heat is generated near the cellular population of interest. Nevertheless, the LEDs can be scaled more effectively than the waveguides in terms of reducing their size and increasing their number, with the potential for highly confined stimulation at multiple locations. In addition, μLEDs consume less power than commercial laser diodes and therefore will be more easily powered wirelessly, which is ideal for behavioral animal experiments (assuming the cost is practical). Both approaches can offer advantages for particular optogenetic applications; however, significant engineering efforts are still needed to achieve their full potential in neuroscience and possible clinical applications.

Fortunately, the field of nanophotonics has developed many approaches to integrating light sources and efficiently transmitting and modulating light. Optogenetics will benefit greatly from these rapid advances, and several groups have already provided evidence that light stimulation arrays are capable of matching the density of electrical recording technology. With greater effort in microsystem design, advanced packaging, and easy-to-use software interfacing, optoelectrode technologies will transition from being used in a few neuroscience laboratories to widespread use.

## Scalable implantable microsystems

Current state-of-the-art electrical recording systems are still far from matching nature’s scale in the central nervous system. The mouse brain has approximately one neuron in every 22 μm voxel^74^, which only passive electrodes can match in 2D, but certainly not in 3D. Although electrical interfaces have been noticeably bad at achieving spatial scale, the resolution and breadth of their temporal domain continues to make this mode very attractive to neuroscientists. The average action potential is ~2 ms long and shows spiking (periodicity) at approximately 5 Hz, although this spiking could be in the range 0.5 and 500 Hz (Ref. 177). A 1 kHz bandwidth can capture most details of a cell, from the single neuron to population activity. Neuroscientists often prefer an ~0.1–10 kHz bandwidth with sub-microvolt analog-to-digital converter (ADC) resolution so that one may analyze the waveform shape itself. Even at a 10 kHz bandwidth, the digital clock speeds in a typical smartphone could sample ~100 000 electrode channels with excellent fidelity.

The scaling bottleneck continues to be the spatial limits of the analog front-ends and the electrode array itself. The first significant limitation of recording systems is the sheer number of interconnects required. When the shank width increases beyond 50–80 μm, there is a noticeable loss of neural signals, thus making this range of widths a widely accepted upper limit. However, as few as 128 channels on one narrow shank pushes the resolution toward expensive manufacturing options such as e-beam or deep ultraviolet lithography. Even if resources were unlimited and e-beam lithography, for example, could be used to pattern the interconnects, the next bottleneck becomes the bonding pad interface, whether it be a cable, PCB, or an ASIC. Recently, one group demonstrated the feasibility of e-beam interconnects resulting in a 1000 microelectrode array, but the structure was still limited by having a very large silicon backend and PCB^178^. We believe the recent advancements in packaging and mixed-mode circuit design are creating a unique opportunity that will accelerate efforts to address scale. We explore some of the latest research that is addressing these limitations, including alternative analog front-end architectures and packaging.

### High-density active recording

Because the vast majority of neurons we have access to are just above the noise floor (~5–20 μV), the strategy of integrating on-chip amplifiers is an attractive one. Low-noise amplifiers (LNAs) on the same substrate as the electrode array (or packaged very close to it) will in theory reduce the load capacitance and attenuation of the biological signal. Short leads also reduce inter-channel crosstalk and become less susceptible to electromagnetic interference. Ideally, the analog front-end density should match that of the electrode array density. Furthermore, if the area spanned by the electrodes could even include the ADC and data serialization circuitry, then what would limit its scalability? If this challenging proposition could be achieved, then the field would have an unprecedented active system—one that is truly expandable and not limited to choosing only a few regions. We discuss recent analog-to-digital systems that have made impressive gains in area efficiency and some of the performance tradeoffs that should be addressed.

**Figure 6:**
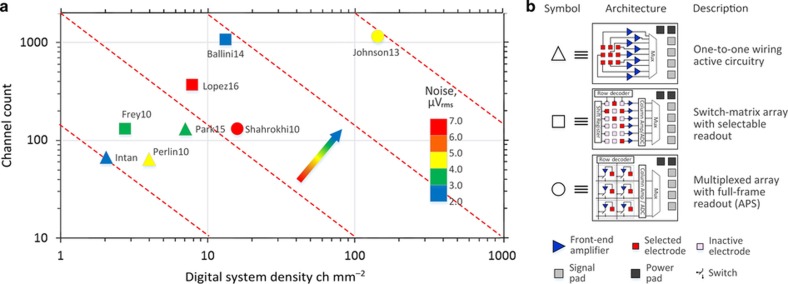
Scalability of leading high-density recording systems having integrated digital output. (**a**) Channel count versus density for three different architectures. Color indicates the input-referred noise (μVrms). Actual channel count was significantly lower compared with the number of available recording sites for the switch-matrix architecture (□). The arrow indicates the direction and color of advancing microsystems. (**b**) Legend for inset A showing three common architectures discussed in recent work. Table 1 draws further comparison.

A comparison of the highest-density and highest-performing systems is shown in Figure 6. Inclusion in this comparison requires that the ASIC integrate the digital convertors with the analog-front end and be capable of wideband recording. Although MEA amplifiers and *in vivo* amplifier ASICs have historically been considered different device types, we wanted to directly compare the best area efficiency from each camp. It should also be noted that the lines between *in vivo* and *in vitro* are blurring because the ASIC can become the probe. A large project undertaken by researchers at HHMI and Imec has recently resulted in an implantable ASIC with 966 selectable channels by post-processing CMOS circuits using a silicon-on-insulator 130 nm process^179^. ASICs may also be further post-processed into flexible arrays, using deep reactive ion etching, that stop on several pre-defined metal etch stops to leave only islands of interconnected electrodes and amplifiers^180^. Other ASICs we include (Figure 6, Table 1) are designed for *in vivo* use and packaged on the backend of the system (intantech.com, Park15 (Ref. 181), and Perlin10 (Ref. 182)). This contrast also allows for the comparison of three different architectures (Figure 6b): (i) one-to-one mapping of electrode to a high-gain LNA, (ii) multiplexed array with a switch matrix to allow site-selective multiplexing, and (iii) a multiplexed array with full-frame readout similar to an active pixel sensor (APS) imager.

Table 1 compares many of the performance metrics of each example as well. It is also critical to note that the many performance metrics can shift in weight based on the application. It can be argued that the emphasis on system density (Figure 6) should not overlook the other metrics. The common-mode rejection ratio, for example, is an important predictor of noise immunity when using a system in awake behaving animals. Obviously, a wireless or battery-powered system places a premium on low power; therefore, the advantages and disadvantage of each approach should be considered in context. Table 1 shows a variety of tradeoffs. The commercial Intan system is currently the lowest-cost solution and exhibits excellent noise performance, but at some cost to size and power. Park15 has been optimized for the combination of low power, high resolution, and moderate density. Lopez16 (Ref. 179) successfully implemented a channel selection switched matrix as the neural probe itself with many competitive metrics. Ballini14 (Ref. 183) and Johnson13 (Ref. 184) achieved the two highest densities with competitive input-referred noise values, but these devices were tested over a higher frequency band, limiting a proper comparison. Nonetheless, the magnitude of the system density suggests that future systems may choose scale over the slight limitation of a smaller bandwidth tuned only for single-unit activity (for example, 300–5000 Hz).

Regarding the noise performance of the various systems, the noise amplitude is somewhat crude because it is not uniformly tested and is a function of both the bandwidth and the actual band. For CMOS devices in particular, flicker noise or 1/*f* noise is the dominant source at low frequencies (corner frequency is typically 500–10 000 Hz) for small transistors. Input referred noise can be skewed by a limited bandwidth and particularly by a large high-pass frequency such as 20 Hz (Table 1). A few important points are worth noting in the model for the flicker noise voltage spectrum *S*^2^:

(5)S1/f2(f)=KfCoxA1fα

where *K*_*f*_ is a technology feature size and is a foundry-specific value, *C*_ox_ is the gate oxide capacitance and *A* is the area of the gate. The exponent *α* is another empirically measured value and determines the slope of the low-frequency response (typically close to 0.9 for NMOS and 1.1 for PMOS)^185,186^. A large *α* will attenuate the noise faster with respect to frequency and thus is desirable. *C*_ox_ is the normalized capacitance, which generally increases with decreasing feature size but will vary depending on the oxide thickness of a given process. Although most LNA circuit designers tend to choose large process nodes (180–600 nm, Table 1), there has been a slow but steady trend toward advanced nodes. One group demonstrated very low noise in a 65 nm node that also had a small amplifier area^187^ of 0.013 mm^2^, although with only two channels. Circuit designers should also pay close attention to the process-defined values of *K*, *C*_ox_, and *α*, which will help reduce noise. Even if transistors must be made much larger than required by the technology node for an equivalent noise floor, the digital circuit design in particular will benefit from the higher density and lower power of a smaller node. Future solutions may also employ the mixing of processes by vertical integration or by special processes such as graphene or SiGe BiCMOS. SiGe BiCMOS can employ low-noise bipolar transistors in the front-end and high-resolution low-power CMOS in the digital domain.

Given these size-performance constraints related to flicker and thermal noise, the strategy employed by the switch matrix architectures (Figure 6) is to separate amplification stages such that a smaller circuit is near the electrode. The electrode stage amplifier is switched into a column readout amplifier, which generally is a programmable gain amplifier (PGA) connected to an ADC. It has been a precept by many that having a dedicated LNA, PGA, and ADC per channel would provide the lowest noise design, but the noise performance of a few switched-matrix MEAs has become good enough that the density advantages may now be attractive for *in vivo* use. To date, very few integrated microsystems have been tested in animals; thus, further *in vivo* validation is needed to assess biological noise and motion artifact noise. The switched matrix design also reduces the number of interconnects leading to the array itself from (row count×column count) to only slightly more than (row count+column count). But the selectable^179,183,188^ and the active pixel architectures^184,189^, have important differences between them.

Using a switched matrix architecture with selectable electrodes (Table 1) is an effective way to reduce power in a circuit design. One can switch the data stream to regions of high activity and ignore quiescent regions. However, an important limitation of this architecture can be illustrated in the context of a clinical application such as a brain-machine interface versus brain mapping. In the former, the clinician can scan for *a priori* features in the neural data, but in the latter, almost nothing is *a priori*. Once a putative neuron signal is found, it is especially important to look across the entire array in search of network effects—activity occurring immediately before and after the action potential to help determine coherence, phase, and ideally, network connections. Selective spike detection as a means of data compression is a challenging and risky proposition for circuit mapping because when one threshold event occurs (a putative neuron), one must simultaneously analyze many or all of the channels on the array or risk losing valuable data. For a high-density array, there will be a good probability of one neuron firing at almost any moment in time, and thus, sampling sporadically is only useful when much is already known. Therefore, high-density full-frame readout schemes such as the ‘active pixel’ approach will ultimately be most attractive to systems neuroscientists.

### Packaging developments

The packaging requirements for implantable and wearable neurotechnology are generally to protect the tissue from heat, to protect the electronics from the ingress of water and ions, and to minimize the device dimensions while presenting a user-friendly form factor. A variety of solutions to these problems have already been pioneered using microfabrication methods to package gyroscopes, infrared sensors, and resonators in very difficult non-biological environments. These applications require atmospheric, thermal, and mechanical isolation and thus provide neurotechnologists with many useful strategies. Similarly, advancements in the vertical stacking of ICs, also known as system-in-package, are providing new methods of packaging to address the needs of combining dissimilar technologies such as MEMS, CMOS ASICs, and memory. Several reviews such as (Refs. 190 and 191) cover a range of creative methods for achieving ultrahigh-density packaging. We will discuss recent work that has borrowed from these two broader packaging developments and have been applied to neurotechnology.

Commercially available recording and stimulating technologies rely on relatively low-density methods; hence, the typical backend of a silicon neural probe consumes the largest area on a given mask set. Wire bonding is the most common method for connecting to a silicon device, and typical bonding pad pitches are 100–150 μm. For polymer substrates, MicroFlex interconnection (a form of ball bumping) offers an alternative to wire bonding but has a similar pitch^192^. Certain high-end consumer products are capable of 40–50 μm pitch, and state-of-the-art packaging research has demonstrated 10 μm pitch using microbumps and flip chip bonding^193^. Unfortunately, these approaches also require greater investment in packaging tools such as die bonders with highly precise thermocompression and alignment capabilities. Finally, the testing tools that ensure reliability will also become more costly with increasing scales and finer features.

Nonetheless, research continues to demonstrate promising solutions. In one example of neural probe-to-ASIC integration, Xie *et al.* improved the encapsulation performance of a wireless microsystem flip-chip mounted on a Utah-style array. Al_2_O_3_ was deposited by atomic layer deposition on both the passive recording array and the integrated backend to significantly extend the lifetime of both compared with the use of parylene-C alone^194^. In another example, Perlin *et al.* demonstrated 3D stacking of high-density planar arrays that were interconnected on a common platform through the novel use of tabs with a pitch of 40 μm (Ref. 182; Figure 1j). Consumer electronics have also created a demand for the stacking of silicon devices. Vertical feedthroughs in an IC die allow the circuit to be stacked on other ICs or on an interposer with multiple ICs in parallel (also known as 2.5D technology). This technology could become a cost effective way to integrate neural probes and front-end amplifiers. Through-silicon-vias (TSVs) have now matured to the point that TSVs within interposers or stacked ICs have demonstrated high yield by a number of vendors. TSVs can also be built into the exterior of a sensor capsule^195^, similar to the way feedthroughs are built into medical devices. MEMS-based solutions for feedthroughs and hermetic capsules reduce the volume of a microsystem by several orders of magnitude compared with conventional titanium housings with ceramic feedthroughs. Furthermore, wafer-level packaging of sensor arrays and vertical enclosures have been demonstrated using a variety of low-temperature eutectic bonds^196^ and thus also have the potential to be cost-effective and compatible with many materials.

Packaging is a significant roadblock to the implementation and commercialization of high-density microsystem tools for neuroscience. The field will continue to witness both monolithic and hybrid solutions in the coming years. Arguably, monolithic integration greatly simplifies the packaging challenge, but as the substrate materials continue to vary and new modalities arise, the field will undoubtedly benefit from the ongoing advancements in heterogeneous vertical stacking. It is not difficult to imagine a day in the near future when vertical IC/MEMS integration is as simple as wire bonding is today. When this happens, we envision that the number of recording and stimulation channels on current ASICs will not necessarily increase, but instead, technologists will modularly package them in parallel on sensor and actuator arrays. In other words, the typical path forward is to move the arrow on the channel count versus density plot (Figure 6) directly orthogonal to the figure of merit, but there might be an advantage if chipsets feature a low channel count and therefore are easily distributed throughout the system. CPU speed and transistor count, by analogy, are no longer steadily increasing; instead, computer makers have relied on parallel processing. With this in mind, hybrid packaging approaches appear to be a practical solution for addressing the need for scalability while acknowledging that applications such as brain mapping and medical devices are inherently small-volume.

## Future technologies for neuroscience research

Since 1990, over one thousand neuroscience and neural prostheses studies have used silicon-based neural probes for monitoring brain activity, and an increasing number using both light stimulation and recording arrays in tandem are being published. Although these advancements are impactful, both neuroscientists and technologists agree that our current rate of development is insufficient to expect major breakthroughs in neuroscience without improved scale, density, and specificity. The goal of reverse engineering even a basic brain function, such as memory or learning, may be many years away given that our tools are still very crude compared with the systems under study. There are no trivial solutions because sensors, actuators, ASICs, and packaging must all progress in step if practical solutions are to become readily available to the larger neuroscience community. The field has recently witnessed an influx of new ideas and researchers with continued government and foundation investment anticipated. We can expect nascent technologies to become increasingly relevant and disruptive.

Optical methods of stimulating and monitoring neural activity have already become an important complement to electrical methods^197^. Future neuroscience discovery will undoubtedly rely on a novel combination of sensing and actuating modalities, but which will be most effective at revealing neuronal cell types and circuit function? Neurochemical sensing and control is arguably the most promising modality yet to achieve scale. Importantly, multimodal methods are needed to combine intracellular monitoring, such as monitoring protein or metabolic changes, with our current extracellular techniques. Although studying the electrical response of a cell in the context of behavior has been important, some experiments will additionally require monitoring intracellular changes, for example, RNA and protein changes^198^, if we are to answer that most basic question: What makes any one neuron fire or not fire? This vision, we believe, will require long-term effort by MEMS and microsystems engineers working closely with neuroscientists. With further interest and investment, we expect the coming decade to see significant breakthroughs in our understanding of brain function and disease-related dysfunction.

## Figures and Tables

**Table 1:** Performance comparison of leading system architectures

	Perlin10	Intan	Park15	Frey10	Ballini14	Lopez16	Shahrokhi10	Johnson13
Architecture, Figure 6b	One-to-one (Δ)			Selectable (□)			Active pixel (○)	
Sampling rate, kHz	16	30	25	20	20	30	14	20
Input noise, μVrms	4.8	2.4	3.3	3.0	2.4	6.4	6.1	4.3
Bandwidth, Hz	10–10k	0.1–10k	0.4–11k	1–100k	300–10k	0.3–10k	10–5k	20–50k
Total power/Ch, μW	—	830	19	1107	73	49	19	—
CMRR, dB	—	—	60	—	72	>60	60	21 (66)^a^
ENOB/resolution	—/8	—/16	10.9/1^b^	—/8	—/10	—/10	6.2/8	8.2/10
No. of Ch. availability	64	64	128	122	1024	384	128	1120
No. of electrodes	64	64	128	11 011	26 400	966	128	1120
Tech node, nm	500	—	180	600	350	130	350	180
Reference	182	c	181	188	183	179	189	184

Abbreviations: Ch, channel; Tech, technology.

a CMRR measured to be 21 dB, but after principal component analysis was performed, 66 dB was achieved.

b ADC oversampled 32X.

c www.intantech.com.
